# CD4 T cell dynamics shape the immune response to combination oncolytic herpes virus and BRAF inhibitor therapy for melanoma

**DOI:** 10.1136/jitc-2021-004410

**Published:** 2022-03-25

**Authors:** Galabina Bozhanova, Jehanne Hassan, Lizzie Appleton, Victoria Jennings, Shane Foo, Martin McLaughlin, Charleen ML Chan Wah Hak, Emmanuel C Patin, Eva Crespo-Rodriguez, Gabby Baker, Edward Armstrong, Matthew Chiu, Hardev Pandha, Adel Samson, Victoria Roulstone, Joan Kyula, Richard Vile, Fiona Errington-Mais, Malin Pedersen, Kevin Harrington, Masahiro Ono, Alan Melcher

**Affiliations:** 1 Translational Immunotherapy/Targeted Therapy Teams, The Institute of Cancer Research, London, UK; 2 Imperial College London, London, UK; 3 Leeds Institute of Medical Research at St. James’s, University of Leeds, Leeds, UK; 4 Radiotherapy & Imaging, The Institute of Cancer Research, London, UK; 5 Institute of Cancer Research, London, UK; 6 Oncology, University of Surrey, Guildford, UK; 7 Molecular Medicine, Mayo Clinic, Rochester, Minnesota, USA; 8 Division of Radiotherapy and Imaging, The Institute of Cancer Research, London, UK

**Keywords:** Oncolytic Virotherapy, Melanoma, Immunomodulation, T-Lymphocytes

## Abstract

**Background:**

Combination herpes simplex virus (HSV) oncolytic virotherapy and BRAF inhibitors (BRAFi) represent promising immunogenic treatments for BRAF mutant melanoma, but an improved understanding of the immunobiology of combinations is needed to improve on the benefit of immune checkpoint inhibitors (ICI).

**Methods:**

Using a BRAF^V600E^-driven murine melanoma model, we tested the immunogenicity of HSV/BRAFi in immunocompetent C57BL mice. In addition to standard FACS analysis, we used the ‘Timer of Cell Kinetics and Activity’ system, which can analyze the temporal dynamics of different T cell subsets. This immune data was used to inform the selection of ICI for triple combination therapy, the effects of which were then further characterized using transcriptomics.

**Results:**

Adding BRAFi treatment to HSV improved anti-tumor effects in vivo but not in vitro. Immune characterization showed HSV or dual therapy led to fewer intratumoral Treg, although with a more activated phenotype, together with more effector CD8 +T cells. Tocky analysis further showed that HSV/BRAFi dual treatment reduced the Tocky signal (reflecting engagement with cognate antigen), in both Treg and conventional subsets of CD4+, but not in CD8 +cells. However, a higher percentage of Treg than of conventional CD4 +maintained frequent engagement with antigens on treatment, reflecting a predominance of suppressive over effector function within the CD4 +compartment. The only T cell subset which correlated with a reduction in tumor growth was within Tocky signal positive conventional CD4+, supporting their therapeutic role. Targeting CD25 high, antigen-engaged Treg with a depleting anti-CD25 ICI, achieved complete cures in 100% of mice with triple therapy. Transcriptomic analysis confirmed reduction in Foxp3 on addition of anti-CD25 to HSV/BRAFi, as well as increases in expression of genes reflecting interferon signaling and cytotoxic activity.

**Conclusions:**

Combination HSV/BRAFi is an immunogenic therapy for BRAF mutant melanoma, but cannot fully control tumors. Dual therapy results in changes in T cell dynamics within tumors, with relatively maintained antigen signaling in Treg compared with conv CD4+. Antigen-engaged CD4 +effectors correlate with tumor growth control, and depletion of Treg by addition of an anti-CD25 ICI, releasing suppression of conventional CD4 +effectors by Treg, enhances survival and activates immune signaling within tumors.

## Background

Treatment for advanced melanoma has dramatically improved over recent years with the introduction of immunotherapy (particularly immune checkpoint inhibitor (ICI) antibodies against PD-1, PDL-1 and CTLA-4), and drugs targeting the MAPK/ERK signaling pathway (BRAF and MEK inhibitors).^1^ However, a significant number of patients do not respond to these interventions, due to the lack of targetable gene mutations (only approximately half of melanomas are BRAF mutated), and/or an unsupportive tumor mutational burden and immune landscape to sustain therapeutic antitumor immune activation with immune checkpoint blockade. Therefore, there remains a need to identify effective additional combination and novel strategies to widen the patient population who will benefit from these interventions. However, the biological understanding of how such further combinations might be optimized, and the responsible mechanisms for effective therapy, remain poorly understood.

A promising candidate combination partner to improve immunotherapy for melanoma is oncolytic viruses (OV), such as herpes simplex virus (HSV). Talimogene laherperepvec (T-VEC), a genetically modified type I HSV encoding granulocyte-macrophage colony-stimulating factor, is clinically approved for intratumoral injection in melanoma, and early clinical data suggested that the virus could convert immunologically ‘cold’ tumors to ‘hot’, and thus prime for effective immune checkpoint blockade.^2^ There is also preclinical evidence to show that the immunomodulatory effects of MAPK/ERK pathway-targeting drugs can enhance OV-based and other immunotherapies, including our own data in a model of BRAF mutant thyroid cancer.^3–8^ In a melanoma model, a MEK inhibitor/HSV combination was dependent on CD8 +cells and Batf3 +dendritic cells (DC), but not CD4 +cells or macrophages, while BRAF inhibitor (BRAFi)/HSV in our thyroid model was dependent on CD8 +and NK cells.^7^ Hence there is evidence for the role of the immune system in HSV/targeted drug combination therapy, though mechanisms remain unclear. Nevertheless, a consistent finding has been that, when HSV was combined with either BRAF or MEK inhibition, the addition of ICI further enhanced therapy, although again the mechanistic rationale underlying the choice of a particular checkpoint molecule acting on a specific effector T cell subset, was not fully defined.^7 8^ An improved biological understanding of the immune consequences of targeted drugs and OV treatments may provide novel mechanistic insights to inform the development of additional interventions to further enhance antitumor immunity.

One important area in the interaction between a tumor and the immune response is the temporal progression and dynamics of T cell activity, which is likely to be critical in shaping the overall T cell response. To address this gap in our understanding, the ‘Timer of Cell Kinetics and Activity’ (Tocky) system was developed, which can analyze the temporal dynamics of antigen-reactive conventional CD4 +Foxp3-, Treg (CD4 +Foxp3+) and CD8 +T cells (online supplemental figure 1).^9 10^ While Tocky has provided insights into aspects of T cell subset dynamics biology, from the perspective of Foxp3 expression as well as antigen engagement, it has not been applied to cancer immunotherapy. Tocky is a reporter system using a fluorescent ‘Timer’ protein, which spontaneously changes its emission from blue to red within 4 hours. Since the red-form protein is stable, this unique maturation allows analysis of rapid temporal changes using transgenic mice, in one strain of which the fluorescent Timer protein is driven off the Nr4a3 promoter (designated as Nr4a3-Tocky), which is faithfully induced by TCR signals. While resting T cells in these mice do not express the Timer protein, once T cells recognize antigen and receive TCR signals, they initiate Timer transcription, and become Blue +Red- (‘new’ cells). T cells with persistent TCR signals sustain Timer transcription over time, and accumulate both blue- and red-form proteins (‘persistent’ cells). When T-cells are removed from their antigens, Timer transcription is arrested, and the unstable blue-form protein is rapidly lost. Then T-cells become Blue-Red +area (‘arrested’ cells). In addition in Nr4a3-Tocky mice, GFP is driven off the Foxp3 promoter, allowing differentiation in TCR signaling between Foxp3- conventional/effector, and Foxp3 +Treg CD4+cells, and additional markers can be used to further characterize the new, persistent and arrested cells. This allows the building of a full mechanistic picture including the dynamic elements of TCR engagement, to enhance both our biological understanding of treatment effects, and direct future therapeutic strategies. In this study, we sought to explore T cell antigen engagement dynamics, alongside established measures of immune cell number, phenotype and activation status, to better define the mechanisms of potentially successful combination therapy with HSV and BRAFi in melanoma. Using this approach, we were able to identify and use addition of a depleting anti-CD25 antibody (aCD25) directed against Treg, to improve prolonged survival to complete cure on addition of aCD25 to HSV/BRAFi double combination therapy.

10.1136/jitc-2021-004410.supp1 Supplementary data

## Methods

### Cell lines

The BRAF-mutant (BRAF^V600E^) mouse melanoma cell line 4434 was established from C57BL/6_BRAF +/LSL-BRAFV600E; Tyr::CreERT2+/o.^11^ Human melanoma cell line Mel888 (BRAF^V600E^) were obtained from the Cancer Research UK cell bank. Cells were cultured in Dulbecco’s modified Eagle’s medium DMEM, supplemented with 10% (v/v) Fetal Calf Serum (FCS) (Thermo Fisher Scientific), 1% (v/v) L-glutamine and 0.5% (v/v) Pennicillin/Streptomycin.

### Small molecule inhibitor, OV and anti-CD25 antibody

PLX4720, a BRAF^V600E^ mutant-specific inhibitor (BRAFi), was obtained from 3way Pharm and dissolved in DMSO. HSV is a HSV type 1, which was kindly provided by Dr Joe Conner (Virttu Biologics, UK). The oncolytic HSV used in this study, HSV1716 (used in the clinic as Seprehvir), has been described previously.^12^ In brief, this HSV is derived from HSV strain 17+with deletions of both copies of *ICP 34.5* gene. A variation of this HSV encoding firefly luciferase (HSV-Fluc) was also used in this study. The titer of each stock was determined by plaque forming assays using Vero cells.^13^ An FcγR-optimized anti-CD25 Treg depleting antibody clone PC61 (aCD25-m2a), as previously described, was used and produced by Evitria (Switzerland).^14^

### Proliferation, survival and viral growth assays

To determine the sensitivity of murine and human melanoma cells to PLX4720 (BRAFi) or HSV, 2×10^3^ cells/well were plated in white polystyrene 96-well plates (Thermo Fisher Scientific) and incubated for overnight at 37°C. Cells were then treated with a range of inhibitor/virus concentrations in triplicates for 72 hours. PLX4720 range was 100 µm–0.005 µm for all cell lines. HSV was diluted at a range of 9–0.004 MOI (Multiplicity of Infection) for all cell lines. DMSO or medium-only wells were included as a control for inhibitor and virus, respectively. Cell survival was measured using CellTiter-Glo Luminescent Cell Viability Assay Kit kit (Promega), represented as a normalized percentage of treated wells to DMSO/medium control wells and used to generate dose-response curves. Luminescence was recorded using the SpectraMax Plus 384 Microplate Reader (Molecular Devices LCC). For in vitro combination studies, 2×10^5^ cells/well were plated in 12-well plates. Cells were then treated with 0.1 µm or 1 µm PLX4720 and/or HSV at MOI of 0.01, 0.1 and 1 for Mel888 and MOI of 0.1, 1 and 10 for 4434 cells. Virus was added 1 hour following treatment with inhibitors. Plates were fixed after 24 hours and 48 hours by adding cold Trichloroacetic acid (TCA) (10% (w/v) in 100% UF H_2_O) to each well at 1:1 ratio of existing volume and incubated at room temperature (RT) for 1 hour. Plates were then washed with tap water and left to dry overnight. Plates were stained with Sulforhodamine B solution (0.057% (w/v) in 1% acetic acid prepared in UF water) for 30–60 min and subsequently washed with tap water and left to dry overnight. Following scanning, plates were solubilized with 10 mM TRIS base solution (pH 10.5) and rocked for 10 min. Absorbance was recorded at a wavelength of 450 nm using the SpectraMax Plus 384 Microplate Reader. To determine viral replication in infected melanoma cells, 1×10^5^ human/murine melanoma cells/well were plated in 24-well plates. Following overnight incubation, cells were treated with 1 µm PLX4720 and/or HSV at MOI 1 (to ensure infection of all cells) and incubated at 37°C for 2 hours. The media containing the virus and/or BRAF inhibitor was then removed, cells were washed twice with growth media and the growth media or PLX4720 were then replaced. At 4, 24 and 48 hours after infection, cells and media were harvested and frozen at – 80°C. The lysate was then freeze-thawed three times and centrifuged at 13 000 rpm for 5 min at 4°C. Supernatant was serially diluted (1:10) and titred by TCID_50_ assay on Vero cells.

### Animal studies

In all animal studies, 4×10^6^ 4434 cells suspended in 100 µL PBS were injected subcutaneously into the right flank of 6–8 weeks old C57BL/6 mice (Charles River) or Nr4a3-tocky:Foxp3-EGFP mice (C57BL/6 background).^9^ In brief, these mice were generated by insertion of a fluorescent timer reporter gene into the first exon of the *Nr4a3* gene, performed in a knock-in, knock-out manner, ensuring that any functional Nr4a3 protein is not produced by the timer transgene. Tocky mice also express the *Foxp3*-internal ribosome entry site (IRES)-green flourescent protein (GFP) transgene under the promoter for *Foxp3*, allowing identification of Treg cells via green fluorescence detection. After sufficient tumor size was reached (14–20 days), mice were randomized and treated. Mice were treated with 40 mg/kg of PLX4720 (BRAFi) via oral gavage (day 0) or vehicle control (5% DMSO in water) daily throughout the experiment. Following 3 days of oral gavage of BRAFi, mice were injected intratumorally with three doses of 5×10^5^ pfu/mouse of HSV every other day (days 3, 5 and 7), injected in 30 µL volume via a Hamilton syringe. Fc-optimized anti-CD25 (aCD25) antibody was injected into 4434 tumor bearing C57BL/6 mice via intraperitoneal injection at a concentration of 200 µg/mouse in 100 µl 2 days prior to commencement of BRAFi oral gavage (day −2), and once weekly thereafter. Tumors were measured twice weekly and tumor volumes were calculated using the formula: length × width × height (mm) × 0.5236. No toxicity, including significant weight loss, was seen in any treated mice. In survival studies, mice were sacrificed once tumor size reached 15 mm in any dimension. Each experiment was repeated at least twice in independent experiments (with 5–6 mice per group), and data combined. The Kaplan-Meier survival curves were compared using the log-rank (Mantel-Cox) test using Prism Software (GraphPad).

### In vivo imaging

A total of 4434 tumor-bearing mice (3 mice/group) were treated with PLX4720 and/or HSV-Fluc as described above. One day following the last intratumoral virus injection (day 8), mice were injected with 75 mg/kg D-Luciferin - K+Salt Bioluminescent Substrate (Perkin Elmer) and imaged 5 min later using The IVIS Spectrum in vivo imaging system (Perkin Elmer).

### Cell isolation from tissues and flow cytometry analysis

Treated mice were sacrificed at different time points (Day 8 and Day 15) and the tumors dissected and weighed. Tumors were dissociated mechanically using scissors and enzymatically digested in RPMI containing 0.5 mg/mL Collagenase type I-S (Sigma-Aldrich), 0.4 mg/mL Dispase II protease (Sigma-Aldrich), 0.2 mg/mL DNase I (Roche) and 4% Trypsin (0.25% in Tris Saline) for 30 min at 37°C. Following digestion, samples were passed through a 70 µm cell strainer and washed with 10% FCS RPMI supplemented with 5 mM EDTA. Resulting samples were divided in equals parts for each antibody panel set into 96 well round bottom plates and incubated with a mouse Fc blocker (BD Pharmingen) at a concentration of 2.5 µg/mL for 10 min on ice. Samples were then stained with a mix of fluorescently-labeled antibodies and viability dye (eBioscience) for 30 min on ice, protected from light. For intracellular staining, samples were permeabilized with the Foxp3/Transcription Factor Staining Buffer Set (eBioscience) for 30 min on ice. Intracellular antibody mix was prepared in the perm wash solution contained in the kit. Samples were then fixed with IC Fixation Buffer (eBioscience) for 20 min at RT and then resuspended in FACS buffer and stored overnight. Samples were acquired on BD LSR II (BD) the following day and quantified using 123count eBeads Counting Beads (Thermo Fisher). Flow cytometry data was analyzed using the FlowJo software (FlowJ, Oregon, USA) where counts were normalized against tumor weight using the 123count eBeads counting beads.

### Gene expression analysis

A total of 4434-tumor bearing mice (3 mice/group) were treated with a double combination of BRAFi and HSV, a triple combination (addition of anti-CD25 antibody) or vehicle control as outlined previously. Mice were sacrificed on day 5 (48 h post first virus injection) and day 8 (24 hours post third virus injection) and tumors were placed in RNAlater RNA Stabilization Reagent (Qiagen). Tumors were lysed in homogenization tubes (Thermo Fisher Scientific) containing buffer Buffer RLT (Qiagen) using Precellys 24 homogenizer. RNA was then isolated using RNeasy Plus Mini Kit (Qiagen) according to the manufacturer’s instructions. RNA concentration was measured using Qubit Fluorometer system and Qubit RNA BR Assay Kit (ThemoFisher), and 96 ng of RNA was used in the nCounter Mouse Immunology Panel (Nanostring Technologies). Normalization, differential expression, geneset analysis and cell type scoring were performed using NanoString nSolver V.4.0 advanced analysis software. Data presentation used Rstudio V.1.4.1103, R V.4.1, ggplot2 and ComplexHeatmap packages.

### Histology and immunohistochemistry

Tumors from treated mice were dissected and fixed overnight in formalin. These were then processed, embedded and 2 µm sections were prepared on APEX glass slides. Sections from Formalin-fixed paraffin-wax embedded tumor were stained with primary anti-rat monoclonal anti-CD8 antibody (eBioscience, clone 4Sm15as). Slides were scanned and imaged using Hamamatsu Nanozoomer (Hamamatsu Photonics).

### Statistical analysis

All statistical analyses were performed using the GraphPad Prism software (GraphPad Software). For tumor growth comparison between treatment groups, area under curves were used to determine statistical significance. The log-rank Mantel Cox test was used to determine statistical differences between survival groups. For immunophenotyping experiments, differences between treatment groups were analyzed using ordinary one-way analysis of variance/Holm-Sidak multiple comparisons test. When data did not follow a Gaussian distribution, nonparametric Kruskal-Wallis/ Dunn’s multiple comparisons test was used. For FACS and tumor growth/Kaplan-Meier in vivo data as shown in figures, * denotes p<0.05, **p<0.01, and ***p<0.001.

## Results

### Hsv oncolytic virotherapy and BRAF inhibition is an effective combination in vivo, but not in vitro, in murine melanoma

**Figure 1:**
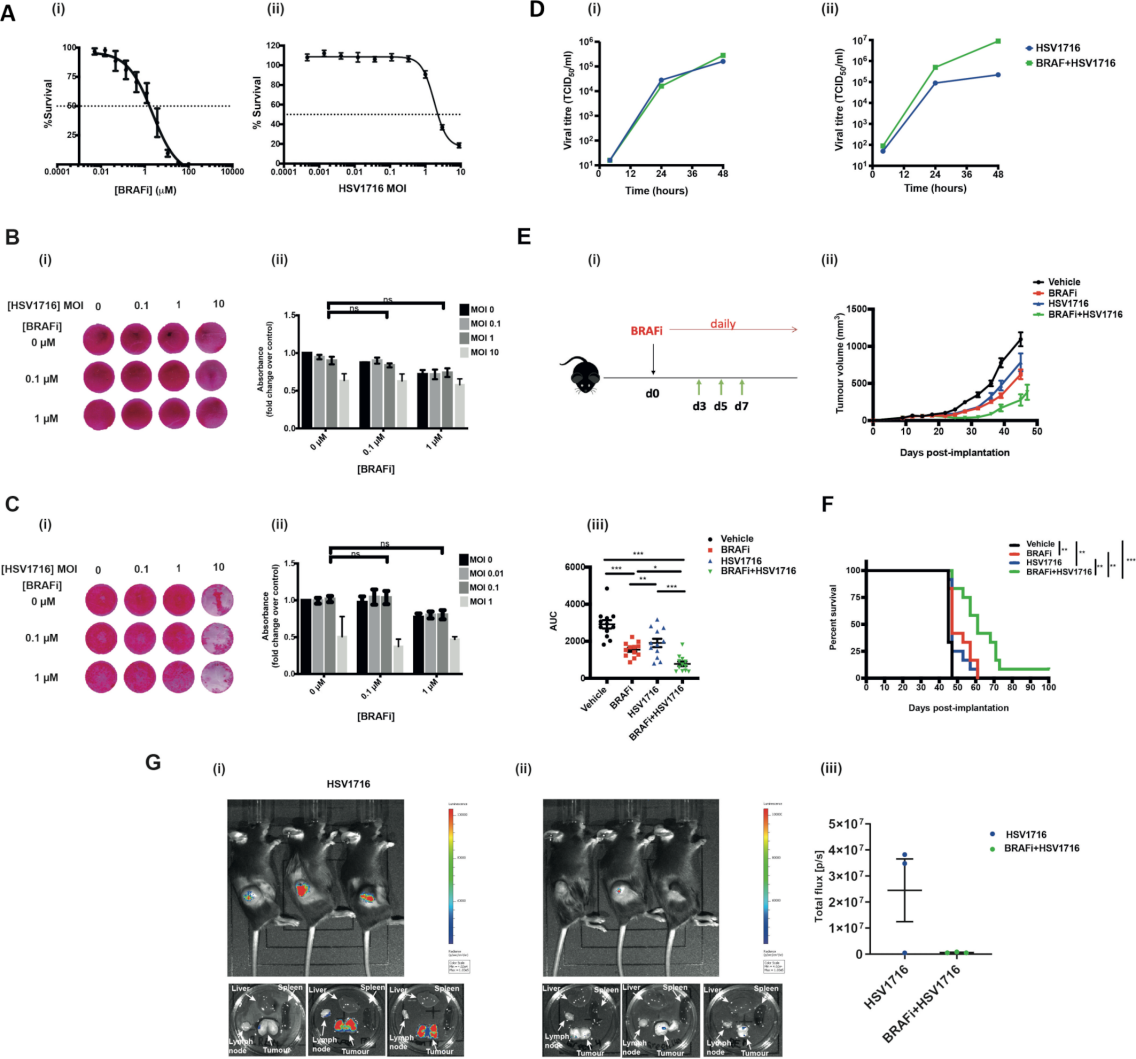
Human and mouse BRAF mutant melanoma cell lines are sensitive to BRAFi and HSV in vitro, but a combinatorial effect is only seen in vivo. (A) 2×10^3^ murine melanoma V600E mutant 4434 cells were seeded in 96-well plates, allowed to adhere and then treated in triplicate with either 100 µm–0.0005 µm of BRAFi (PLX4720) (i) or 9–0.004 MOI of HSV (ii) for 72 hours. Signal was measured using CellTiter-Glo luminescent assay kit. Per cent survival was calculated as a normalized ratio of signal measured in treated wells to the wells treated with DMSO control. 2×10^5^ 4434 (B) or Mel888 (C) cells were plated in 12-well plates and treated with PLX4720/HSV the following day. Plates were fixed and stained 48 hours post-treatment; the experiment was repeated at least three times and the image shown in 2Bi/2 Ci is representative of one experiment. Stained plates were solubilized and assessed for cytotoxicity with a sulforhodamine B (SRB) colorimetric assay. Data in 2Bii/2Cii represents an average of three biological repeats with error bars representing SEM; treatments were compared using two-way ANOVA with multiple comparisons. (D) Virus titer of HSV retrieved from 4434 (i) or Mel888 (ii) cells treated with HSV alone or in combination with PLX4720 at various time points. 1×10^5^ cells were treated with HSV (at an Moi of 1 to ensure infection of all cells)±1 µm BRAFi PLX4720 for 2 hours and the virus was then washed off and replaced with fresh medium containing BRAFi in the combination condition. Cell-free supernatants were used for the analysis, and the viral titer was determined by TC_ID50_. (E) (1) 4×10^6^ 4434 cells were implanted into the right flank of C57BL/6 mice; treatment schematic. (ii) After sufficient tumor growth was reached, on day 0, mice were treated with 40 mg/kg BRAFi via oral gavage for 3 days followed by HSV1716 (5×10^5^ pfu/mouse) intratumorally every other day as indicated by the green arrows. Oral gavage was continued daily throughout the experiment. average tumor volume was calculated by the formula volume=width × length ×height x 0.52. Growth curves represent mean values from 12 mice/group±SEM mice were sacrificed once 15 mm3 of either dimension was reached. (iii) Area under the curve (AUC) was measured up to day 45 post tumor implantation. One-way ANOVA with multiple comparisons test was performed to test for significance. Vehicle vs BRAFi ***p<0.0001; vehicle vs HSV **p=0.0016; vehicle vs BRAFi/HSV p<0.0001; BRAFi vs BRAFi/HSV *p=0.02226; HSV vs BRAFi/HSV ***p=0.0004 F) Kaplan-Meier survival curve. curves were compared using the log-rank (Mantel-Cox) test. vehicle vs BRAFi **p=0.003; vehicle vs HSV **p=0.0022; vehicle vs BRAFi/HSV ***p<0.001; BRAFi vs BRAFi/HSV **p=0.0049; HSV vs BRAFi/HSV **p=0.001 (G) 4×10^6^ 4434 cells were implanted into the right flank of C57BL/6 mice and tumors were grown and treated as described previously for figure 2E (n=3/group). Mice were treated with a combination of BRAFi and HSV1716-Fluc to enable imaging of virus. 24 hours post the third virus injection (day 8), mice were injected with luciferin and imaged for 1 min using the IVIS spectrum in vivo imaging system machine. mice were then sacrificed and their organs dissected and imaged. bioluminescent images of mice treated with (1) HSV1716 alone or (2) BRAFi +HSV1716, (3) quantification of signal expressed as total flux per second. ANOVA, analysis of variance; HSV, herpes simplex virus.

Having previously shown that the combination of HSV oncolytic virotherapy and BRAFi is effective in BRAF mutant thyroid cancer,^8^ we wished to test this approach in the clinically more common setting for use of these drugs, namely melanoma. When BRAFi and HSV were tested in combination in vitro in the BRAF V600E mutant murine melanoma 4434, although both agents were cytotoxic when tested alone (figure 1A), no significant combination effect was seen (figure 1B). The same was seen for the human melanoma cell line Mel-888 (figure 1C). Consistent with this, no significant combinatorial effect was seen for virus replication across mouse and human melanoma lines (figure 1D). Nevertheless, since our thyroid model had shown that the immune response to combination HSV/BRAFi significantly contributes to therapy,^8^ we went on to test the combination in melanoma in vivo. In immunocompetent mice, we found that BRAFi plus HSV was significantly more effective than either agent alone with regard to tumor growth (figure 1E) and survival (figure 1F), although the combination was not curative. To address whether the addition of BRAFi to HSV affected virus replication in vivo, we injected luciferase-expressing HSV into 4434 tumors, and found that the luciferase signal was, in fact, significantly reduced when BRAFi was added, likely due to the toxicity of the drug to tumor cells restricting their ability to support virus infection and spread in vivo (figure 1G). Taken together, these data suggest that the addition of BRAFi to HSV impairs virus infection and replication in vivo, despite enhancing therapy, further suggesting the importance of immune activation over oncolytic cytotoxicity, as the mechanism responsible for the benefit of HSV/BRAFi treatment in this melanoma model.

### Combination HSV and BRAFi increases the activity of both Treg and CD8 T cells in the tumor immune microenvironment

**Figure 2:**
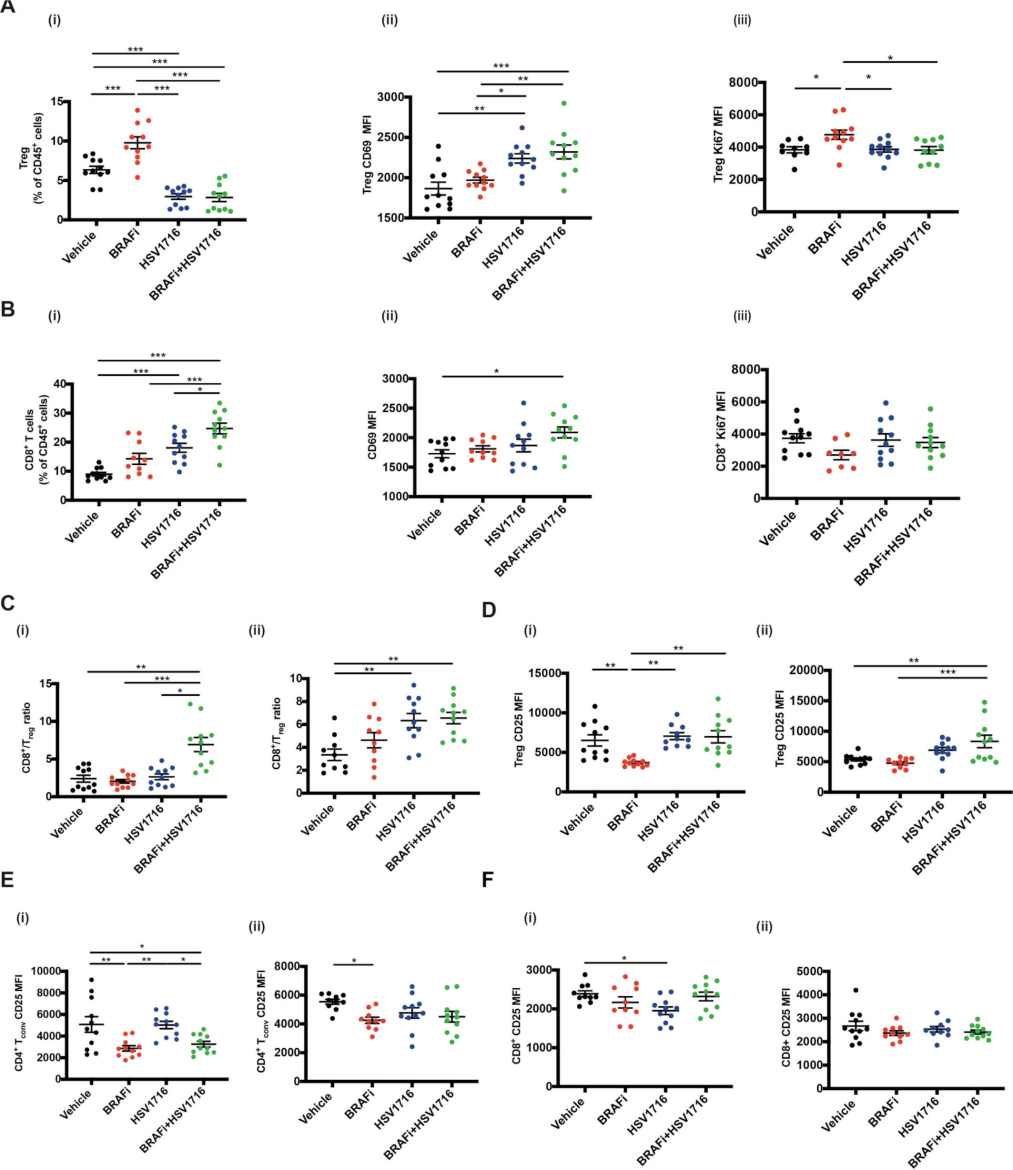
The effect of HSV/BRAFi dual therapy on intratumoral T cell subsets. 4×10^6^ 4434 cells were implanted into the right flank of C57BL/6 mice and tumors were grown and treated as described previously for figure 2E (n=5–6/group). Mice were sacrificed on day 8 or day 15, and whole tumors processed for multi-color flow cytometry analysis. In all experiments, average tumor size at the start of treatment was the same between all treatment groups. Each experiment was performed at least twice with similar results. Each dot represents an individual mouse. Samples were analyzed using one-way ANOVA with multiple comparison test using GraphPad prism software. Data show mean±SEM. (A) Changes in Tregs on day 8 following treatment start: (i) percent CD4 +Foxp3+Tregs out of total immune cell population (all CD45 +cells). Mean fluorescent intensity (MFI) of (II) CD69 and (III) Ki67 on CD4 +Foxp3+Tregs. (B) changes in CD8 +T cells on day 15 following treatment start: (i) percent CD8 +T cells out of total immune cell population (all CD45 +cells). MFI of (ii) CD69 and (iii) Ki67 on CD8 +T cells. (C) CD8 +T cell to CD4 +Foxp3+CD25+Treg ratio in tumors analyzed on (i) day 8 and (ii) day 15 following treatment start. (D) MFI for CD25 expressed on Tregs in tumors analyzed on (i) day 8 and (ii) day 15 following treatment start. (E) MFI for CD25 expressed on conventional CD4 T cells (Foxp3-) in tumors analyzed on (i) day 8 and (ii) day 15 following treatment start. (F) MFI for CD25 expressed on CD8 +T cells in tumors analyzed on (i) day 8 and (ii) day 15 following treatment start. ANOVA, analysis of variance; HSV, herpes simplex virus.

We next characterized the T cell immune infiltration into tumors treated with HSV and BRAFi. Consistent with our previous data in a BRAF mutant thyroid model, we saw significant changes with combination treatment. The percentage of Treg within CD45 +cells decreased with HSV or HSV/BRAFi although, strikingly, their activation state was increased, particularly as assessed by CD69 (figure 2A). In contrast for CD8 +cells, although they were significantly increased as a percentage of CD45 +cells with treatment, there was less clear evidence of any significant change in their activation status, with the only significant change being for dual therapy relative to vehicle (figure 2B). As a consequence of the rise in CD8 +and fall in Treg, the CD8:Treg ratio, often taken as a positive signal of effective immunotherapy, increased with HSV/BRAFi treatment (figure 2C). To further address T cell function, and Treg in particular, we next focused on CD25, which can be used as an additional marker of Treg, but is also a measure of their early activation and suppressive function.^15–17^ CD25 on CD4 +Foxp3+Treg was increased by virus-containing treatment (figure 2D), but reduced on conventional effector CD4 +Foxp3 cells by HSV/BRAFi (figure 2E), and relatively unchanged on CD8+ (figure 2F). This suggests modification of the immune environment on combination treatment, including a shift in the functional balance between suppressive Treg and conventional CD4 cells. While the increase in CD8:Treg ratio on double therapy, which we also saw in our previous thyroid model,^8^ looks favorable, the increase in the activation marker CD25 levels on Treg, relative to effector CD4 and CD8, may limit the effectiveness of therapy.

### Characterization of T cell dynamics of HSV/BRAFi treatment using ‘Timer of cell kinetics and activity’

Treg require T-cell receptor (TCR) signaling for eliciting their suppressive function.^17 18^ We, therefore, sought to go beyond static cell markers of activation status such as CD69, Ki67 and CD25, and directly interrogate the dynamics of T cell signaling, to better understand changes in the immune tumor microenvironment (TME) occurring with HSV/BRAFi therapy. To address this antigen-specific T cell immune response to treatment, we employed the Tocky system.^9 10^ As described earlier, Tocky uses Fluorescent Timer as a reporter gene to analyze transcriptional activities of genes of interest. In this study we used Nr4a3-Tocky, which analyses a gene faithfully induced by TCR stimulation. Since Fluorescent Timer protein spontaneously and irreversibly changes its emission spectrum from blue to red, Nr4a3-Tocky allows analysis of temporal changes in effector cells with a T cell receptor, on their engagement with antigen (online supplemental figure 1). The Tocky system therefore allows us to probe in greater functional detail, the role of different T cell subsets during HSV/BRAFi treatment, focusing on cells with active TCR engagement leading to signaling through Nr4a3.

**Figure 3:**
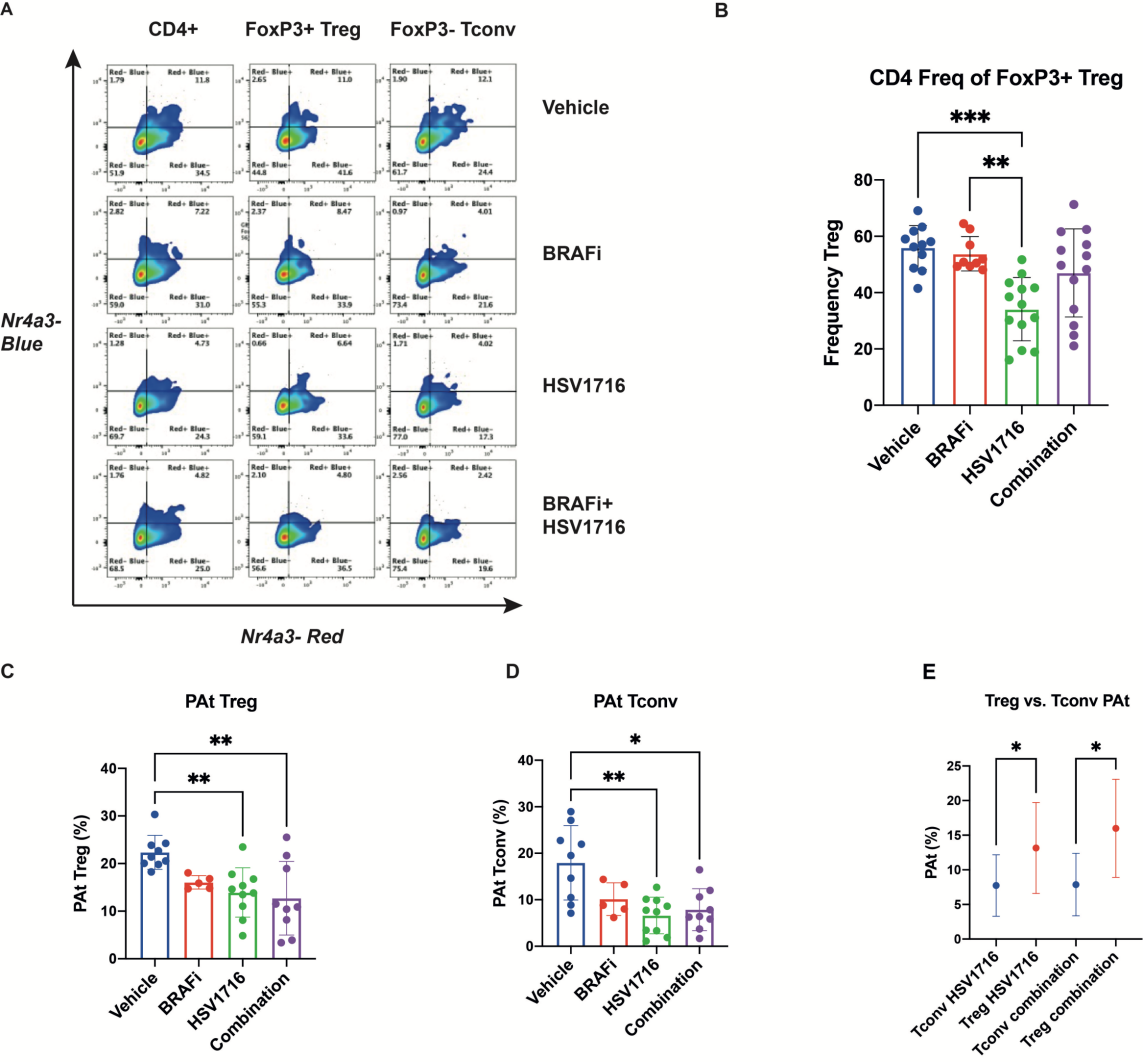
HSV/BRAFi treatment differentially shapes regulatory and effector CD4 TCR signaling dynamics. 4434 melanoma tumor-bearing NR4A3 Tocky mice were treated with single agent BRAFi (PLX4720), HSV (HSV1716) or combination treatment as in figure 2Ei, and analyzed on day 8. NR4A3 blue and red fluoresences was measured in tumor infiltrating lymphocytes by flow cytometry. (A) Representative flow cytometry plots showing NR4A3 blue vs NR4A3 red Tocky fluorescence in total CD4+, CD4 +Treg and CD4 +conv T subsets by treatment. (B) Frequency (%) of Treg (gated as FOXP3 +CD4+cells), within parent CD4 +population by treatment Group. (C, D) Frequency of Persistent-Arrested (PAt) Treg (C), and conv CD4+ (D) populations by treatment Group. (E) Percentage of PAt cells, within their parent population, of conv CD4 +vs Treg in single agent HSV and combination treatment groups. HSV, herpes simplex virus.

**Figure 4:**
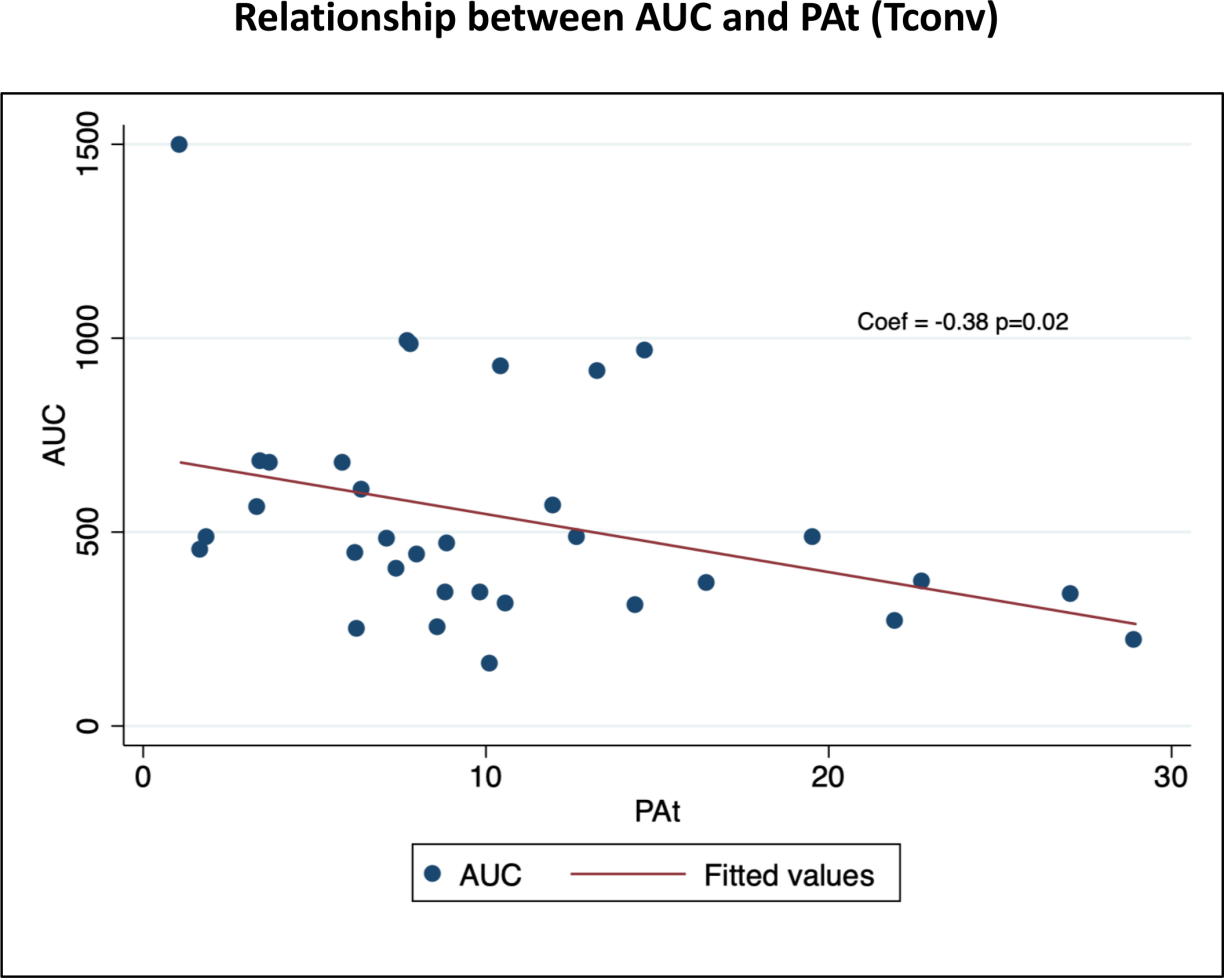
The persistent to arrested (PAt) transitioning Tocky timer signal in conventional CD4 cells correlates with reduction in tumor growth. Spearman correlation analysis between area under the curve (AUC) and PAt cells in conv CD4 +tumor infiltrating lymphocytes independent of treatment group across multiple experiments (n=33 mice).

Tocky was analyzed at day 8, 1 day after the last of 3 intratumoral injections of HSV given on alternate days starting on day 3, and 8 days after the commencement of daily BRAFi (figure 1Ei). At this time point (which matches the day 8 time point in figure 2), significant changes in the Tocky signal were seen in CD4+, but not CD8+ (data not shown), T cells. As expected, in control mice the majority of Treg (CD4 +Foxp3+) were Timer Red+, indicating that they had recognized antigens, which reflects their self-reactive nature and is compatible with our previous findings.^9^ In the absence of treatment, 55.2% of CD4 +Foxp3+T cells were Timer+ (including Blue+Red-, Blue+Red+, and Blue-Red+), whereas 38.3% of CD4 +Foxp3- T-cells expressed Timer (shown in the control vehicle top row, figure 3A). We further analyzed the Tocky locus of each Timer-expressing cell, in which ‘New’, ‘Persistent’ and ‘Arrested’ cells are identified for their transcriptional dynamics of new transcription, persistent transcription, and transcription which has once been activated but arrested, respectively. The two between regions, New-Persistent (NPt) and Persistent-Arrested (PAt) are designated as NPt and PAt (online supplemental figure 1). Generally, consistent with the non-Tocky data of figure 2, this analysis again showed reduction in the number of Treg with HSV treatment, although here this was significant only for HSV monotherapy, but not HSV/BRAFi (figure 3B). Furthermore HSV, with or without BRAFi, treatment reduced the Tocky signal specifically in PAt CD4 cells, both Treg (figure 3C), and conventional (figure 3D), but not in CD8 cells (data not shown). This signal in this Tocky category indicates consistent engagement with cognate antigen, indicative of effector function. However, when the percentage of cells within their parent population retaining their timer positivity was compared between Treg and conventional CD4, it was higher with treatment for Treg (figure 3E). Hence, although treatment reduced the Tocky signal in both effector and regulatory CD4 cells, a higher percentage of Treg maintained frequent engagement with antigens, potentially preventing CD4 effector T-cells from interacting with their antigenic targets. Furthermore, the number of PAt conventional CD4 (but no other T cell subset) correlated with a reduction in tumor growth independent of treatment (figure 4), supporting the therapeutic significance of this Tocky category of effector CD4 cells, even in the non-curative conditions of double therapy. Taken together, these Tocky data indicate that the differential effects of HSV/BRAFi treatment on conventional and Treg CD4 cells, impacts on their TCR signaling dynamics, potentially limiting the efficacy of double combination treatment.

### Targeting CD25 on Treg further improves HSV/BRAFi combination treatment via modification of the immune microenvironment, resulting in tumor cure

**Figure 5:**
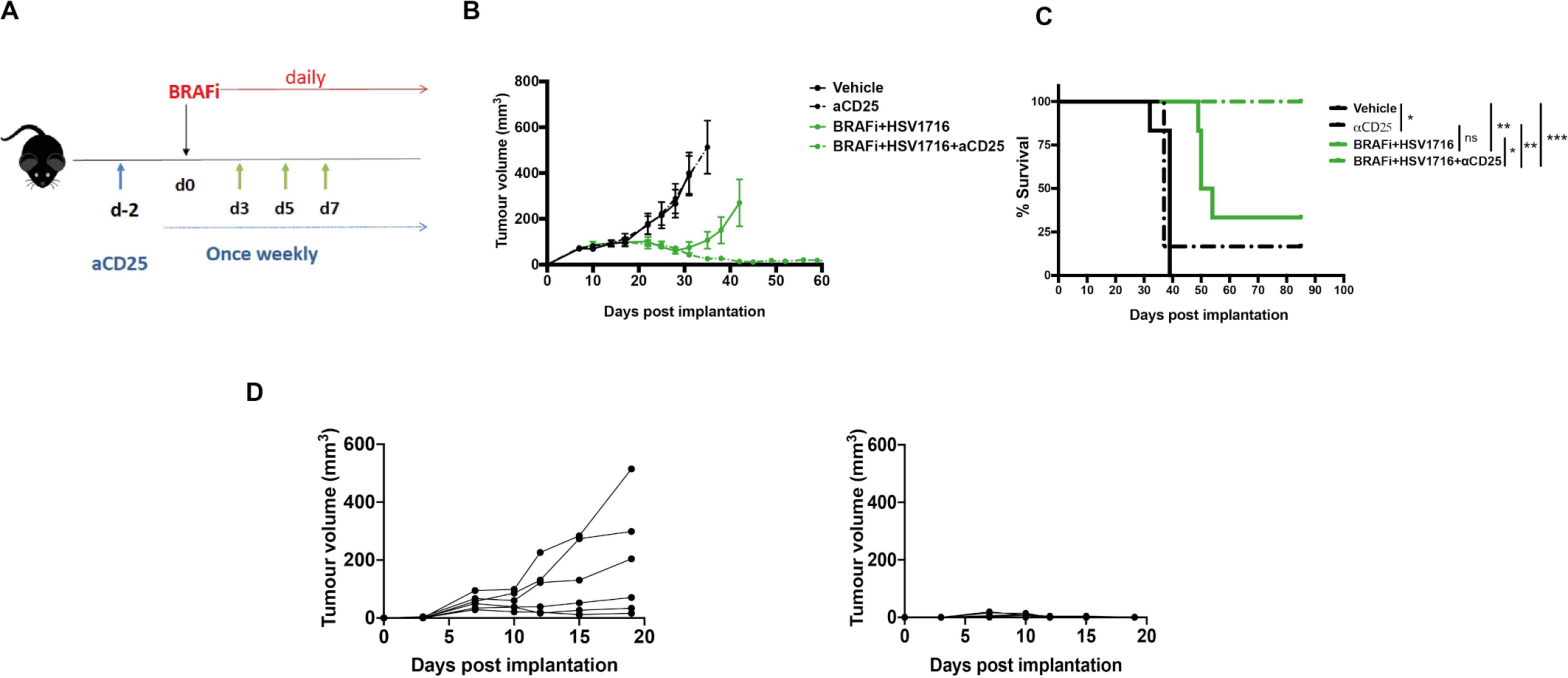
The addition of a depleting anti-CD25 antibody to HSV/BRAFi treatment results in complete cure of tumors. (A) Treatment schematic. A total of 4×10^6^ 4434 cells were implanted into the right flank of C57BL/6 mice. After sufficient tumor growth was reached, on day −2, mice were treated with 0.2 mg/mouse of aCD25 antibody via an intraperitoneal injection. Subsequently, mice were treated as described before: on day 0, mice were treated with 40 mg/kg BRAFi via oral gavage and/or HSV1716 at 5×105 pfu/mouse intratumorally every other day as indicated by the green arrows (n=6 mice/group). In all experiments, average tumor size at the start of treatment was the same between all treatment groups. (B) Average tumor volume. Tumor volume was calculated as described in figure 2. Growth curves represent mean values from 6 mice/group±SEM mice were sacrificed once 15 mm^3^ of either dimension was reached. (C) Kaplan-Meier survival curve. curves were compared using the log-rank (Mantel-Cox) test using prism software. vehicle vs aCD25, *p=0.0255; vehicle vs BRAFi/HSV1716 **p=0.0012; vehicle vs BRAFi/HSV1716/aCD25 **p=0.0012; aCD25 vs BRAFi/HSV1716/aCD25, **p=0,0051; BRAFi/HSV1716 vs BRAFi/HSV1716/aCD25, *p=0.0185. (D) mice with cured tumors following triple combination of BRAFi +HSV1716+aCD25 were rechallenged with 4×10^6^ 4434 cells injected in their contralatera flank (right graph) and the tumor growth was monitored. Six naïve mice served as controls (left graph), and individual tumor growth curves are shown. HSV, herpes simplex virus.

Taken together, the standard FACS characterization of T cells in figure 2, with the Tocky data from figure 3, led us to conclude that tumor-reactive Tocky signal positive PAt Treg, although reduced in number by HSV/BRAFi treatment, become activated and are preferentially engaged with antigens, relative to their effector, conventional CD4 cell counterparts. Moreover, since tumor growth was inversely correlated only with PAt conventional CD4 +cells (figure 4), additional targeting of the Treg remaining after double treatment, might improve the success of therapy. We further hypothesized that the high expression of CD25 on remaining Treg, relative to conventional CD4 +and CD8+T cells (figure 2D–F), may represent a means for improving the T cell dynamic balance between suppressive and effector T cell subsets. Targeting CD25 would preferentially deplete highly active, suppressive Treg over effector conventional CD4 +and CD8+, building on the partial therapeutic success of HSV/BRAFi double therapy (figure 1E and F). Previous studies have shown that a highly effective targeted strategy is to use an Fc-optimized anti-CD25 antibody (with murine IgG2a and k constant regions) to deplete intratumoral Treg.^14^ We therefore tested this antibody as an additional component in a triple combination strategy to target and deplete the remaining highly activated CD25-expressing Treg seen in tumors after HSV/BRAFi double treatment. As shown using the regime in figure 5A, addition of anti-CD25 depleting antibody significantly improved HSV/BRAFi treatment, resulting in tumor control in all mice (figure 5B and C), which were additionally protected from re-challenge, consistent with curative treatment generating long-term antitumor immunity (figure 5D).

**Figure 6:**
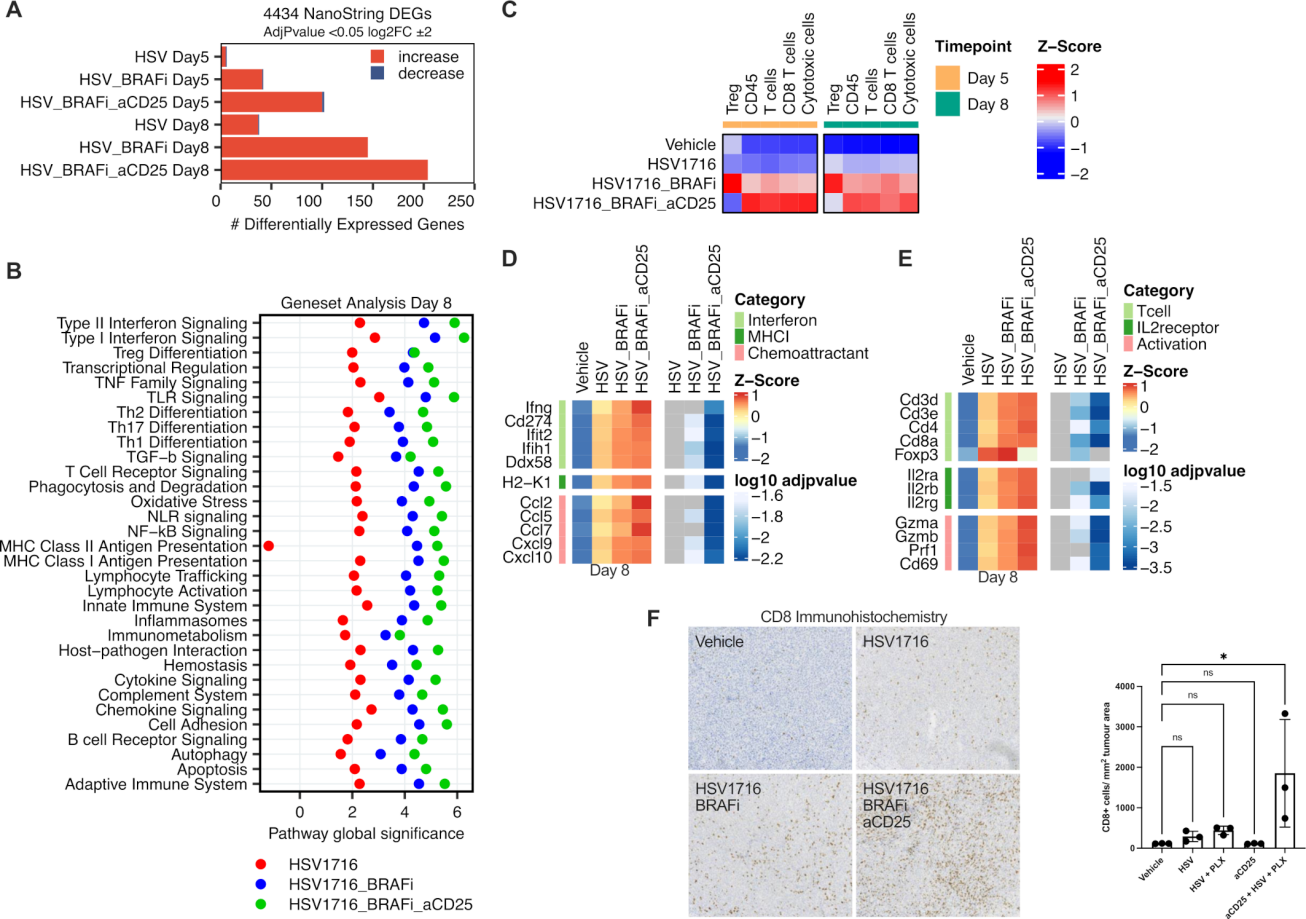
Anti-CD25 depletes Tregs enhancing the inflammatory response and CD8 infiltration in response to HSV/BRAFi. (A) Transcriptomic analysis was performed on tumors at day 5 (day of second HSV injection) and day 8 (the day after the third HSV injection) using the NanoString murine immunology panel. The number of differentially expressed genes (DEGs) was calculated for HSV, HSV/BRAFi, or HSV/BRAFi/anti-CD25 relative to the vehicle only control for each timepoint using nSolver analysis software. (B) Geneset analysis was performed on transcriptomics data using nSolver analysis software. Data shown is the directed global significance statistic, a measure of the tendency of a pathway to have over or under represented differentially expressed genes. positive values indicate upregulation of genes within each geneset. (C) Immune cell type scoring was performed using Solver analysis software. Heatmaps corresponding to interferon and cytokine signaling (D), and T cell, IL-2 receptor and cytolytic activity (E). Data shown are z-scores of log2 transformed normalized counts, plotted alongside log10 adjusted p values for each gene calculated from Deg analysis using nSolver analysis software. (F) CD8 infiltration was assessed by immunohistochemistry at day 15. Quantification was performed (QuPath V.0.3.0), per unit tumor area excluding areas of necrosis, n=3 per group. Mean and SD are shown and normality confirmed with Shapiro-Wilk test, one-way ANOVA with Dunnett’s multiple comparison test. On comparison between vehicle and triple therapy, p=0.0171. ANOVA, analysis of variance; HSV, herpes simplex virus; ns, not significant.

We then compared the transcriptomics from tumors treated with HSV alone, HSV/BRAFi double, or HSV/BRAFi/anti-CD25 triple therapy, using the NanoString nCounter murine Immunology panel. The number of differentially expressed genes under these conditions on day 5 (ie, 2 days after a single HSV injection) and day 8 (the day after the third HSV injection), was greater for double and triple therapy than HSV alone, which caused relatively little disruption to the tumor immune microenvironment (figure 6A). Expression across the immune-focused nanostring panel was increased, with almost no transcripts decreasing, indicative of induction of a proinflammatory environment by treatment, greater for triple than double therapy. Geneset analysis illustrated the immune signaling pathways impacted by treatment. A stepwise increase was observed in the significance scores of all genesets moving from single agent HSV, to HSV/BRAFi, to HSV/BRAFi/anti-CD25 at day 8 (figure 6B), the same time point as the Tocky analysis in figure 3. Comparing triple HSV/BRAFi/anti-CD25 to double HSV/BRAFi, it is notable that Treg differentiation diverged from the pattern of the increases observed for other genesets on addition of anti-CD25, in that it remained unchanged (figure 6B, third row down, blue and green circles). Cell type scoring, comparing triple with double treatment, showed a reduction in Treg (but not CD8 or cytotoxic T cell) population estimates due to anti-CD25-mediated depletion (figure 6C), consistent with the higher CD25 expression on Tregs relative to conventional CD4 and CD8 cells shown in figure 2D–F. To further address the wider consequences of targeting CD25, we focused on key genes reflective of inflammatory markers. At day 8, the addition of BRAFi to HSV clearly increased transcript levels and differential expression of interferon linked genes, MHC class I and chemoattractants (figure 6D), as well as transcripts linked to T-cell populations, the IL-2 receptor, and cytotoxic activity (figure 6E). The addition of anti-CD25 to HSV/BRAFi further increased transcript levels and enhanced p-values associated with differential expression (figure 6D–E). The exception to this pattern was Foxp3, where anti-CD25 clearly decreased transcipts (figure 6E). Similar data for Foxp3 was observed at day 5 (online supplemental figure 2B). There was also an indication that anti-CD25 increased transcripts of genes required for TGF-beta signaling as well as TGF-beta molecules themselves (online supplemental figure 2C), which is of interest and warrants further investigation, given the key role of TGF-beta in inducing Foxp3 expression and peripheral Treg differentiation.^19^ Transcriptomic data also revealed that treatment increased gene expression reflecting myeloid and B cells, as well as Fc receptors, within the TME (online supplemental figure 2D). Finally, the ability of anti-CD25 to enhance the infiltration of CD8 T-cells in response to HSV/BRAFi was confirmed at day 15 after the start of therapy by immunohistochemistry for CD8 +cells (figure 6F). Taken together, this data suggests that the curative addition of anti-CD25 to HSV/BRAFi treatment, transcriptomically depletes Treg and results in a more pro-inflammatory tumor immune microenvironment.

## Discussion

Although treatment for advanced melanoma has improved over recent years, disease is still not controlled in a significant number of patients, and these individuals eventually succumb to their disease. Immunotherapy with ICI and BRAF/MEK inhibitors (in the approximately half of patients with BRAF mutant disease), represent the main drugs in current standard of care treatments. How ICI and BRAFi might be combined has been a relevant clinical question for some time, particularly in light of data that targeted drug therapy can be immunomodulatory and improve responses to immunothery.^3–7^ While early attempts to combine ICI and BRAFi resulted in significant problems with toxicity, more recent early and later stage trials have made progress in overcoming these challenges, with encouraging signs of improved efficacy.^20 21^ A further type of immunotherapy which might enhance ICI/BRAFi dual therapy is oncolytic virotherapy with HSV, an example of which is the clinically approved virus for melanoma, T-Vec. We previously showed, in a model of BRAF mutant thyroid cancer, that HSV/BRAFi was an effective combination, further improved by the addition of ICI as triple therapy,^8^ and here we have further developed this approach in the more common clinical context of melanoma.

Within this study, we wished to improve our understanding of the mechanisms underlying successful combination immunotherapy, with a particular focus on T cell kinetics and dynamics. Immune reactivity can change rapidly over short periods of time, and standard immune readouts measured as correlates of therapy, including the flow cytometry analysis of tumor-infiltrating immune cells and tumor transcriptomics used within this study, are unavoidably restricted to providing a snapshot of the particular time point at which samples are taken. Tocky (Timer of cell kinetics and activity), was developed as a system to move beyond such a constrained temporal focus and address the kinetics of the T cell response, by using a fluorescent timer protein with a short half life, under the control of defined gene promoters. Because the fluorescent Timer protein changes its emission from blue to red over a time frame of only 4 hours (in contrast, the half life of GFP is about 26 hours), and since the red form is stable, Tocky allows analysis of rapid temporal changes in transgenic mice using both timer angle and timer intensity (online supplemental figure 1).^9 10^ In the model used in the current study, the Timer protein is driven by the promoter for the Nr4a3 gene, which is induced by signaling through the TCR, so that the transition in TCR-stimulated T cells from newly activated (blue), through persistent (blue/red or purple) to arrested (red), with NPt and PAt as stages in between, can be accurately quantitated. This allows the T cell dynamics of TCR signaling through new to persistent to arrested (with the additional potential for arrested cells to be reinvigorated to persistent if further TCR signaling resumes), to be assessed in the context of therapy. This can inform and support the role of cells at different stages of Timer fluorescence progression in the success of treatment and, together with analysis of additional markers on Tocky signal positive T cells, such as immune checkpoints, suggest additional targets to improve therapy.

We initially found, consistent with our data in thyroid cancer,^8^ that HSV and BRAFi together were not additive in vitro, but were a effective combination in vivo, supporting a role for the immune response in the benefit of giving these two treatments together (figure 1). However, this dual therapy was still not curative, suggesting that additional immune interventions might improve on the partial success of HSV/BRAFi. Immune characterization by standard flow cytometry of intratumoral T cells showed an increase in the CD8:Treg ratio with HSV/BRAFi but, when cell numbers were taken into account as well as activation markers, an interesting distinction between cell types emerged. Treg numbers fell with dual treatment (figure 2A), but their activation measures increased, while the opposite was the case for CD8 +cells (figure 2B). When CD25 was examined as a further activation marker (with a particularly crucial role in Treg function),^16 17 22^ it again increased on Treg, but in contrast fell on effector CD4 and was relatively unchanged on CD8 (figure 2D–F). It is also worth noting the high relative level of expression of CD25 on Treg, which is lower on conventional CD4, and lower still on CD8. These data suggested that investigation of the dynamics of different T cell subsets using Tocky may provide further insights into which cells may be most functionally important, and potentially targetable.

Inevitably in the context of a dynamic system, the selection of optimal timing for Tocky analysis is uncertain. While we are continuing to explore changes in Tocky in tumors over time in detail (including in the absence of any treatment), in this study we elected to analyze 1 day after the last injection of HSV, to match the day 8 time point of our standard FACS data in figure 2. We did not see any significant differences with treatment in the Nr4a3 Tocky signal in the CD8 +population (data not shown), but there were changes in CD4 +subsets. In particular, significant changes were seen in the persistent to arrested transitioning Tocky signal positive populations, representing cells with consistent effector engagement with cognate antigen. In both Treg and conventional CD4, the PAt Tocky signal fell with HSV/BRAFi therapy (figure 3C and D), although the percentage of timer positive PAt cells remained higher in the Treg population. Hence a higher percentage of Treg remained frequently engaged with antigen with treatment, potentially suppressing and preventing effector CD4 cells from interacting with their TCR targets.

Taking the conventional flow cytometry and Tocky data together, we reasoned that the highly activated Treg, even though reduced in number after dual HSV/BRAFi therapy, were having a functionally important role in limiting the activity of the antigen-experienced conventional CD4+, which contributed to the slowing of tumor growth (figure 4), and that depleting these remaining Treg would further enhance therapy. We developed this further by focusing on CD25, because (1) CD25 was upregulated on Treg (relative to conventional CD4 and CD8), at the same time point of the Tocky analysis (figure 2D–F), and (2) CD25 has been shown to be an effective target for Treg depletion.^14^ Indeed, we found that by adding an optimally depleting anti-CD25 antibody to HSV/BRAFi, we were able to cure 100% of mice, which were subsequently protected from tumor rechallenge (figure 5). It is noteworthy that 4434 is resistant to standard ICI, suggesting that our HSV/BRAFi/anti-CD25 approach may be particularly relevant in checkpoint-refractory disease.

While we would ideally have liked to confirm Treg depletion on the addition of anti-CD25 at the cellular level, we found that triple treated tumors were too small at the necessary, comparative time point, to allow FACS analysis (standard or Tocky), as performed in earlier experiments. We therefore instead analyzed the transcriptome to assess the impact of the addition of anti-CD25 to HSV/BRAFi therapy. This showed induction of a more pro-inflammatory TME with the inclusion of anti-CD25, and cell sorting analysis suggested specific depletion of Treg relative to other T cell subsets (figure 6), consistent with their high CD25 expression (figure 2D). It is noteworthy that genes representing TGF-beta and its signaling were modulated with HSV/BRAFi and HSV/BRAFi/anti-CD25 treatment (online supplemental figure 2C); on day eight half were upregulated and half downregulated. This is consistent with the positive as well as negative immune regulatory roles of TGF-beta and Treg which have been described, and reflect potential upregulation in signaling through pathways important in peripheral Treg differentiation.^19^ However, in the current context, any enhanced signaling may not lead to fully functional Foxp3-expressing suppressive Treg, particularly on inclusion of anti-CD25, due to the Treg depleting effects of the antibody, which instead leads to a TME more favorable for immunotherapy. Together with Tocky data showing that a higher percentage of Treg than conventional CD4 maintained frequent engagement with antigens on treatment with HSV/BRAFi, these data support a paradigm whereby anti-CD25 targets and depletes a functionally highly suppressive population of Treg signaling through their TCR.

One interesting question arising from our data was the dependence of triple therapy on immune cell subsets as assessed by cell depletion experiments. While in our thyroid model we found HSV/BRAFi therapy to be significantly dependent on CD8 (but not CD4) cells,^8^ in this melanoma model we saw no significant difference in HSV/BRAFi/anti-CD25 treatment benefit with CD8 + or CD4+ depletion (data not shown). While we are currently testing different timings of depletion to explore this further, one potential explanation, supported by our data, is that cell depletion experiments, particularly of CD4 cells (which cannot differentiate between Treg and conventional CD4), may have complex effects on the functional interactions between different T cell subsets. Variations between different models, dual vs triple therapy, and choice of ICI, are likely also important, but Tocky highlights how depletion experiments may not provide appropriate data to fully inform on mechanisms underlying immunotherapy.

In summary, we have shown that HSV/BRAFi is a promising dual therapy for melanoma, which is further improved by the addition of a depleting anti-CD25 antibody. Dual HSV/BRAFi therapy had a differential effect on the T cell dynamics of different CD4 +subtypes, such that persistent to arrested transitioning TCR signaling was maintained in a higher percentage of activated Treg than conventional CD4, and these PAt CD4 effectors were the T cell subset which correlated with reduction in tumor growth. Using Tocky to provide data on the dynamics of TCR signaling between different T cell subsets in relation to therapy, alongside flow cytometry characterization of targetable molecules on particularly suppressive T cells, directed us towards a rational, mechanistically informed triple combination HSV/BRAFi/anti-CD25 strategy, supporting its progression to clinical testing.

## Conclusions

Our data show, for the first time, how the dynamics of T cell antigen signaling can be tracked in tumors, including in the context of treatment. Dual combination HSV/BRAFi was immunogenic as assessed by standard methods such as the CD8 to Treg ratio, but Tocky analysis revealed that only the persistent to arrested transitioning Tocky positive TCR-signaling subset of conventional CD4 +effectors correlated with reduced tumor growth. Additional targeting of CD25 high Treg, which maintained their TCR signaling to a greater extent on treatment than conventional CD4+, with a depleting anti-CD25, improved HSV/BRAFi therapy by releasing inhibition of CD4 +effectors and enhancing immune activation within tumors. Hence, characterization of T cell signaling dynamics can provide important information about subsets responsible for successful cancer immunotherapy and, alongside other measures of targetable molecules, inform the choice of additional drugs to improve treatment.

## Data Availability

All data relevant to the study are included in the article or uploaded as online supplemental information. Not applicable.
