# Supplementary figures and images for: CD4 T cell dynamics shape the immune response to combination oncolytic herpes virus and BRAF inhibitor therapy for melanoma
Source: J Immunother Cancer. 2022 Mar 25;10(3):e004410. doi: 10.1136/jitc-2021-004410 (PMC8961178; doi:10.1136/jitc-2021-004410)

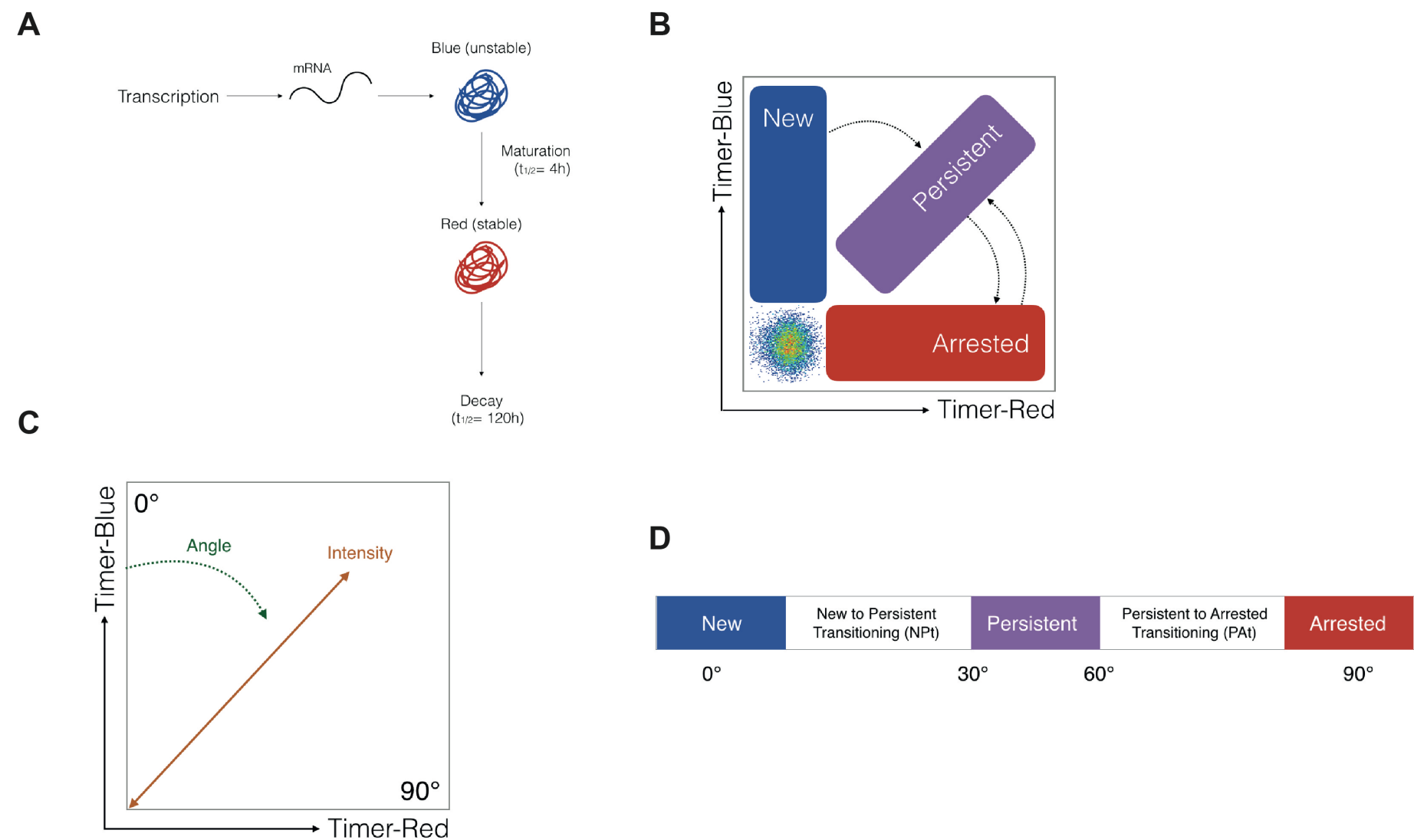

Supplementary Figure 1

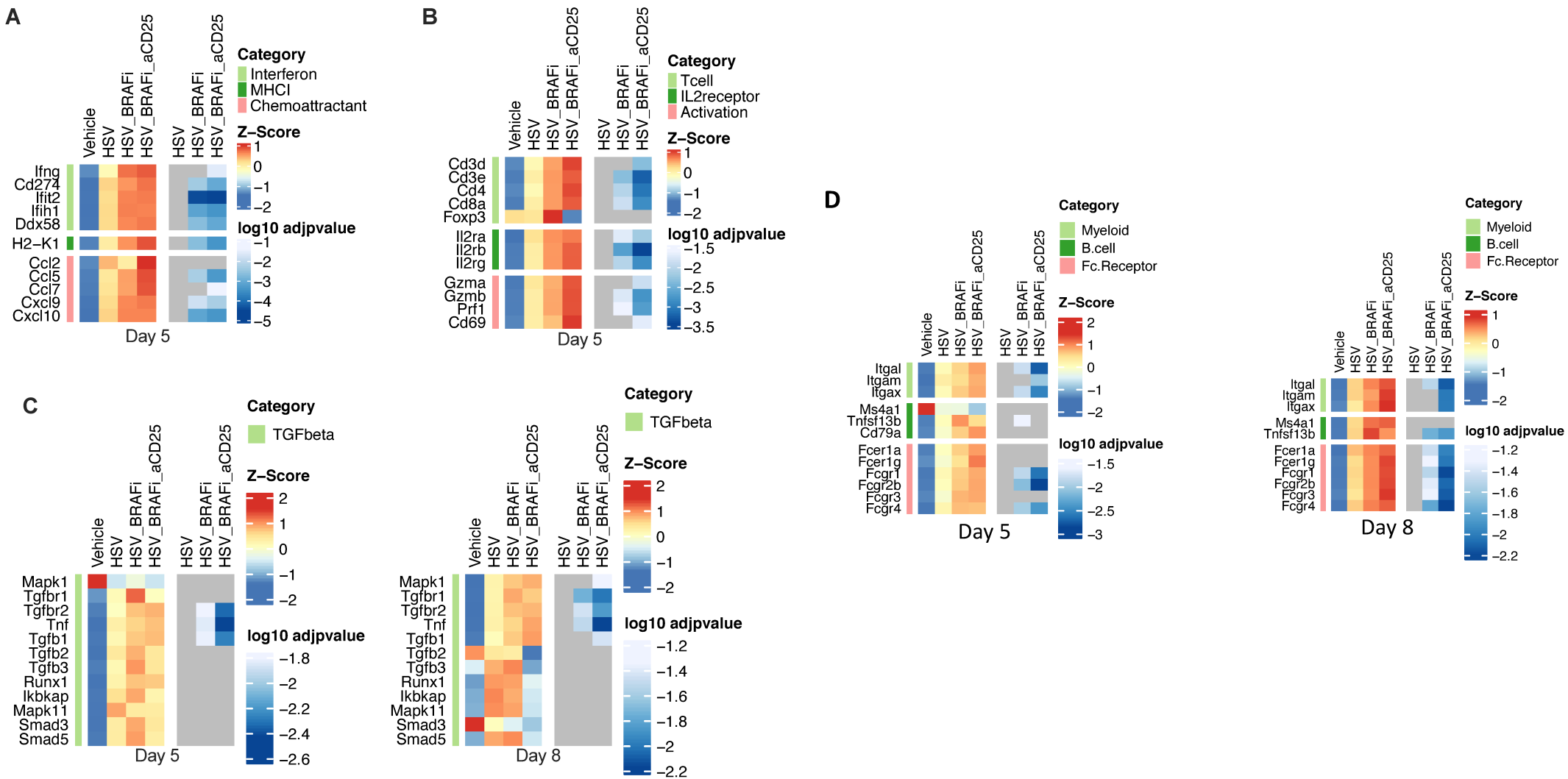

Supplementary Figure 2

Supplement: Supplementary data [file jitc-2021-004410supp001.pdf]
